# Differential Effects of Aging on Regional Corpus Callosum Microstructure and the Modifying Influence of Pulse Pressure

**DOI:** 10.1523/ENEURO.0449-23.2024

**Published:** 2024-05-14

**Authors:** Jessica N. Kraft, Stephanie Matijevic, David A. Hoagey, Kristen M. Kennedy, Karen M. Rodrigue

**Affiliations:** ^1^ Center for Vital Longevity, Department of Psychology, School of Behavioral and Brain Sciences, The University of Texas at Dallas, Dallas, Texas 75235; ^2^Department of Psychology, University of Arizona, Tucson, Arizona 85721

**Keywords:** aging, corpus callosum, diffusion imaging, pulse pressure, vascular risk, white matter

## Abstract

The corpus callosum is composed of several subregions, distinct in cellular and functional organization. This organization scheme may render these subregions differentially vulnerable to the aging process. Callosal integrity may be further compromised by cardiovascular risk factors, which negatively influence white matter health. Here, we test for heterochronicity of aging, hypothesizing an anteroposterior gradient of vulnerability to aging that may be altered by the effects of cardiovascular health. In 174 healthy adults across the adult lifespan (mean age = 53.56 ± 18.90; range, 20–94 years old, 58.62% women), pulse pressure (calculated as participant's systolic minus diastolic blood pressure) was assessed to determine cardiovascular risk. A deterministic tractography approach via diffusion-weighted imaging was utilized to extract fractional anisotropy (FA), radial diffusivity (RD), and axial diffusivity (AD) from each of five callosal subregions, serving as estimates of microstructural health. General linear models tested the effects of age, hypertension, and pulse pressure on these cross-sectional metrics. We observed no significant effect of hypertensive diagnosis on callosal microstructure. We found a significant main effect of age and an age–pulse pressure interaction whereby older age and elevated pulse pressure were associated with poorer FA, AD, and RD. Age effects revealed nonlinear components and occurred along an anteroposterior gradient of severity in the callosum. This gradient disappeared when pulse pressure was considered. These results indicate that age-related deterioration across the callosum is regionally variable and that pulse pressure, a proxy of arterial stiffness, exacerbates this aging pattern in a large lifespan cohort.

## Significance Statement

Aging is associated with microstructural changes in the corpus callosum, the largest white matter tract in the brain. Additionally, cardiovascular factors, such as hypertension and pulse pressure, affect corpus callosum microstructure. However, it is unclear whether these factors uniformly impact the corpus callosum throughout aging. The current study aimed to characterize patterns of corpus callosum aging and assess the influence of hypertension and pulse pressure across subregions of callosal white matter. We found an age-related gradient on corpus callosum microstructure, with the most pronounced impact on anterior regions. However, this gradient was absent when pulse pressure was considered. These findings suggest that callosal subregions are differentially sensitive to age-related decline, and pulse pressure modifies and exacerbates these declines.

## Introduction

The corpus callosum (CC) subregions are characterized by distinct axonal diameters, fiber densities, and myelination levels (Aboitiz et al., 1992). Projections from sets of homologous cortical areas traverse the callosal subregions (Hofer and Frahm, 2006), enabling each subregion to facilitate distinct aspects of cognition (Baird et al., 2005). Specific subregions reach maturation at different points in development (Lebel et al., 2010) and each callosal subregion may be differentially vulnerable to aging (Michielse et al., 2010).

MRI-based diffusion techniques (e.g., diffusion tensor imaging; DTI) allow for indirect assessments of white matter (WM) microstructural health. DTI evaluates axonal attributes of WM via information on the orientation and magnitude of water diffusion (Basser and Pierpaoli, 1996). Fractional anisotropy (FA), which measures the directionality of diffusion, varies across the callosum (Ota et al., 2006; Hasan et al., 2009), reflecting differences in axonal composition. Nonlinear associations between FA values and age exist, including increases in FA from birth to young adulthood and decreases through adulthood (McLaughlin et al., 2007; Hsu et al. 2010). Age-related losses in FA in healthy adults may be greatest for anterior WM regions and least severe for posterior regions (Bennett et al., 2010) suggesting an anteroposterior deterioration gradient of aging (Head et al., 2004). This gradient likely mirrors the developmental pattern of CC subregions, referred to as the “last in, first out” principle (Raz and Kennedy, 2009; Brickman et al., 2012).

Additional DTI metrics, including radial diffusivity (RD) and axial diffusivity (AD), are thought to indicate more specific forms of WM damage than that captured in FA (Metwalli et al, 2010). Animal research suggests that increases in RD may represent de/dysmyelination, whereas increases in AD may represent Wallerian degeneration and total axonal loss (Song et al., 2003). Typically, nonpathological brain aging is associated with increases in RD and minimal-to-no increase in AD, though the literature is mixed (Bender and Raz, 2015).

Along with age, cerebro- and cardiovascular health influence WM integrity. Insufficiency of brain perfusion inordinately affects WM compared with gray matter (Pantoni et al., 1996), likely due to vasculature density differences: capillary density in WM is six times lower than in gray matter (Kubíková et al., 2018). In individuals with dementia, this discrepancy is larger, reduced by 18%, and strongly reduced in individuals poststroke by 57% (Hase et al., 2019). Small vessel disease is one of the most prevalent neurological conditions, arising from arteriolosclerosis, commonly caused by hypertension (for review, see Pantoni 2010). Approximately 40% of Alzheimer's disease (AD) patients show vascular pathology postmortem (Boyle et al., 2018). Individuals diagnosed with hypertension at midlife evidence a 24% higher risk of developing dementia (Whitmer et al., 2005), and ideal blood pressure (BP) is one of the biggest protective factors against subclinical silent brain infarcts (Gardener et al., 2018).

Hypertension, specifically, is associated with a higher incidence of WM hyperintensities and greater age-related reductions in anterior CC FA (Burgman et al., 2010). Greater baseline cardiovascular risk (including hypertension) is associated with substantial longitudinal declines in posterior CC FA (Williams et al., 2019). Given that even incremental elevations in blood pressure in healthy adults negatively affect WM microstructure (Maillard et al., 2012; Hoagey et al., 2021), blood pressure metrics closely link cardiovascular system health to the brain, thus providing a sensitive measure for studies of aging. The pulse pressure index (PP) incorporates heart ejection fraction and arterial wall pliability (Al Rifai and Al-Mallah, 2018) and thus serves as a proxy for arterial stiffness (Safar, 2018). PP is a reliable risk factor for cardiovascular disease in both normotensive and hypertensive populations, even after adjusting for other cardiovascular risk factors (e.g., smoking, cholesterol levels; Thomas et al., 2013; Boutouyrie et al., 2021).

The current study seeks to fill the gaps in the literature on the contributions of cardiovascular health to WM aging. While older age and hypertensive risk factors have been linked to the degradation of callosal integrity (Williams et al., 2019), the intersection of these risk factors and their spatial pattern throughout adulthood remains unclear. Therefore, the study goal was to investigate how two cardiovascular health indices (hypertension, arterial stiffness) moderate the aging of CC WM health across its segments. We hypothesized poorer FA, RD, and AD in (1) older adults, (2) individuals with hypertension/ higher pulse pressure, and (3) older adults who have hypertension or higher pulse pressure (age–pulse pressure/hypertension interaction). We further hypothesized (4) an age-related anteroposterior gradient along the CC segments (akin to retrogenesis in other brain regions) and (5) that this gradient will be diminished with cardiovascular risk (i.e., more posterior segments also affected).

## Materials and Methods

### Participants

Participants included 174 healthy, right-handed, native English-speaking adults aged 20–94 (102 women, 72 men) who were recruited for the study through advertisements and flyers from the local community and were compensated for their participation. Participants were screened to be free from cardiovascular disease, diabetes, cancer, neurological and psychiatric disorders, and drug and alcohol abuse through both phone and mail-in health questionnaires prior to entry into the study. Participants also underwent screenings for vision, hearing, and cognitive impairment at the first study visit. Those with a score of >16 on the Center for Epidemiological Studies Depression Scale (Radloff, 1977) or a score of <26 on the Mini-Mental Status Exam (Folstein et al., 1975) were considered ineligible. Written informed consent was obtained in accordance with the guidelines set by local institutional review boards. Participants underwent two cognitive testing sessions and an MRI session. See Table 1 for participant demographic information.

**Table 1.** Sample demographics and blood pressure measurements

Demographic characteristics	
Number of participants	174
Age, mean ± SD	53.56 ± 18.90
Sex *N* (Male/Female)	72/102
Years of education, mean ± SD	15.50 ± 2.50
MMSE, mean ± SD	29.02 ± 0.85
CESD, mean ± SD	4.32 ± 3.79
BMI, mean ± SD	27.30 ± 5.13
Smokers, *N* (%)	7 (4.07%)
High cholesterol, *N* (%)	36 (20.93%)
Hypertensives, *N* (%)	38 (21.84%)
Systolic BP, mean ± SD	126.37 ± 16.54
Diastolic BP, mean ± SD	75.26 ± 9.54
Heart rate, mean ± SD	68.43 ± 9.84
Pulse pressure, mean ± SD	51.11 ± 12.06
Mean arterial BP, mean ± SD	92.29 ± 10.93

MMSE, Mini-Mental Status Exam; CESD, Center for Epidemiological Studies Depression Scale; BMI, body mass index; BP, blood pressure.

### Blood pressure measurements

Participants provided information on hypertension diagnoses and antihypertensive medication use during the initial screening process. Systolic blood pressure (BP), diastolic BP, and heart rate readings were collected during two cognitive testing sessions and the MRI session. Readings for all three visits were averaged and used to calculate mean pulse pressure (systolic minus diastolic BP), which is indicative of arterial stiffness in large arteries. Participants with a previous diagnosis of hypertension by a physician were classified as diagnosed hypertensives, while participants with either mean systolic BP ≥ 140 mmHg or mean diastolic BP ≥ 90 mmHg and no previous hypertension diagnosis were classified as undiagnosed hypertensives.

### Neuroimaging acquisition and preprocessing

All participants were scanned on the same 3 T Philips Achieva MR scanner (Philips Medical Systems) with a 32-channel head coil using SENSE encoding. A 3D T1-weighted MP-RAGE image was acquired with a single turbo field echo sequence (160 sagittal slices, TR = 8.3 ms, TE = 3.8 ms, flip angle = 12°, T1  = 1,100 ms, voxel size = 1 × 1 × 1 mm^3^, acquisition time = 3:57 min). Diffusion-weighted (DW) scans were obtained using a single shot echoplanar imaging sequence (65 axial slices, 30 gradient directions, *b* = 1,000, one non–diffusion-weighted b_0_, TR = 5,611 ms, TE = 51 ms, flip angle = 90°, voxel size = 2 × 2 × 2.2 mm^3^, slice thickness = 2.2 mm, acquisition time = 4:19 min).

T1 images were skull stripped (via BET; Smith, 2002), intensity bias corrected, and registered to Montreal Neurological Institute 1 mm template space (Montreal Neurological Institute, McGill University) via ANTS (Avants et al., 2011). Diffusion images were visually inspected for scanner artifacts and brain abnormalities. Automated software was used to detect motion, susceptibility, and eddy current distortions, while corrections were applied by either using a linear registration of each diffusion gradient to the non–diffusion-weighted b_0_ or removing corrupted gradients from analysis via DTIPrep (Liu et al., 2010). Diffusion directions were adjusted to account for the reorientation of individual gradients (Leemans and Jones, 2009). DTI scalar and tensor maps were calculated using the DSI Studio software program (Yeh et al., 2013).

### Callosal segmentation and fiber tracking

**Figure 1.**
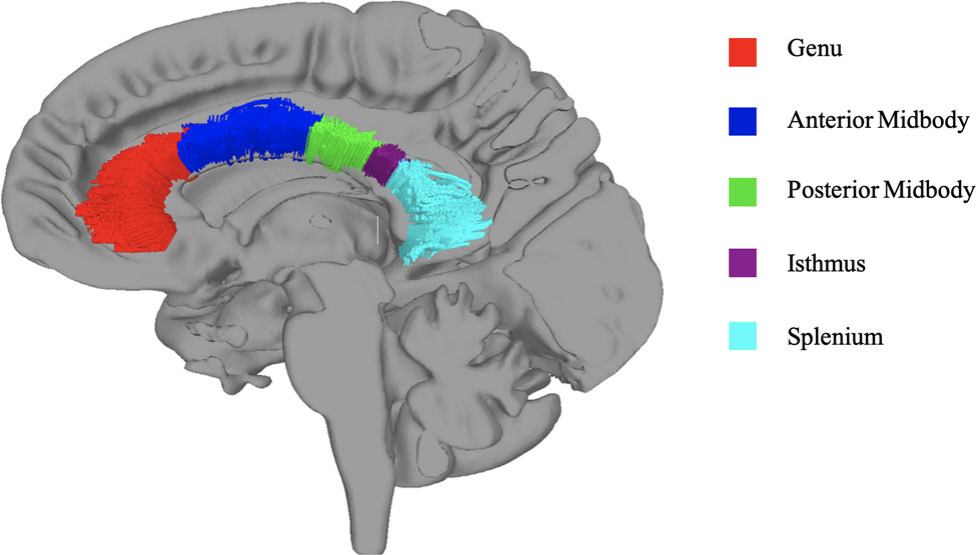
Example of callosal subregion ROI tracts registered to a subject’s native *b*_0_ image. Color represents each callosal subregion tract fiber.

Diffusion indices were extracted from callosal segments using an approach that elucidated white matter fibers via deterministic tractography within a region of interest (ROI) defined as the corpus callosum. Using previous parcellation schemes as a guide, the corpus callosum was manually traced on the 1 mm MNI template to create five separate topographic subregions of genu, anterior midbody, posterior midbody, isthmus, and splenium (cf. Witelson 1989; Hofer and Frahm 2006). Nonlinear registration algorithms placed each callosal division in the participant's native diffusion space to define the region with which to restrict tractography results (Avants et al., 2011). Interhemispheric projections were resolved via streamlined deterministic tractography using the midsagittal plane as an ROI to restrict tracking (Yeh et al., 2013). Additionally, to remove extraneous fibers projecting into the cingulum, we isolated the cingulum bundle separately for use as an exclusion mask. The resulting fibers were divided into the five callosal subregions based on their location within the manually traced ROIs. This approach was used to ensure that diffusion metrics would only be extracted from viable fibers, which were both interhemispheric and within a subdivision of the corpus callosum. Mean fractional anisotropy (FA), radial diffusivity (RD), and axial diffusivity (AD) were extracted from each of the five callosal segment tracts. See Figure 1 for an illustration of the tractography-guided segmentation results.

### Statistical analysis

To test the hypotheses, a series of general linear models (GLM) were computed. In all models, the dependent variable was the multivariate factor consisting of the 5 CC segment tract diffusion metric, with a series of models for FA, RD, and AD, independently. The first three models were run to examine age effects on each of the diffusion properties, with age (continuous, mean-centered) as a between-subjects factor and CC_segment as a within-subjects factor, as well as CC_segment–age interaction. These analyses allowed us to test for differential age effects across the corpus callosum segments and examine a differential effect along the anteroposterior gradient (Hypotheses 1 and 4). The other six models tested cardiovascular risk: three models were specified as above but, in addition to age, included pulse pressure (continuous, mean-centered) and its between- and within-subjects interactions and three models with hypertension status (categorical), age, and all interactions. These models allowed us to test whether pulse pressure or hypertensive status was associated with FA, RD, and AD for each corpus callosum segment, whether there is an age–hypertension/pulse pressure interaction, and whether pulse pressure or hypertensive status alters the anteroposterior gradient (Hypotheses 3, 2, and 5, respectively). Nine models in total were conducted (cf. Tables 2–4). Significant interactions involving continuous variables were decomposed using the Johnson–Neyman (Preacher et al., 2006) and simple slope techniques. Comparisons across correlational effects were tested using Steiger's *Z* (e.g., diffusion metric–age association among the CC segments). In the first specification of the models, sex was included as a covariate of no interest, but showed no reliable effects and was removed to conserve statistical power. Similarly, model reduction was performed when between-subjects higher-order interaction terms were nonsignificant (Pedhazur, 1997). Of note, one individual was excluded from RD analyses for extreme outliers, defined as having a value of >3 times the interquartile range (IQR) in each subsegment of the CC. One additional participant was excluded from AD analyses within the genu of the CC as an extreme outlier, defined as a value >3 times the IQR.

**Table 2.** Regression model for age

Metric	Predictor	df, df_error_	SS	F	*p*
FA	Between subjects				
	Age	1, 171	0.347	77.867	<0.001***
	Within subjects				
	Subregion	4, 684	1.395	476.799	<0.001***
	Subregion x age	4, 684	0.046	15.577	<0.001***
RD	Between subjects				
	Age	1, 171	1.123	105.901	<0.001***
	Within subjects				
	Subregion	4, 684	2.217	395.324	<0.001***
	Subregion x age	4, 684	0.129	22.963	<0.001***
AD	Between subjects				
	Age	1, 171	0.215	10.239	0.002**
	Within subjects				
	Subregion	4, 684	1.970	159.027	<0.001***
	Subregion x age	4, 684	0.072	5.818	<0.001***

df, degrees of freedom; SS, sum of squares; FA, fractional anisotropy; RD, radial diffusivity; AD, axial diffusivity.

*p < 0.05, **p < 0.01, ***p < 0.001.

**Table 3.** Associations between age and DTI metrics within CC segment tracts

Segment	FA		RD		AD	
	Mean ± SD	*r*	Mean ± SD	*r*	Mean ± SD	*r*
Genu	0.63 ± 0.04	−0.628**	0.49 ± 0.06	0.694**	1.56 ± 0.07	0.387**
Anterior Midbody	0.63 ± 0.04	−0.612**	0.50 ± 0.07	0.677**	1.60 ± 0.07	0.359**
Posterior Midbody	0.65 ± 0.05	−0.515**	0.48 ± 0.07	0.561**	1.64 ± 0.09	0.136
Isthmus	0.63 ± 0.06	−0.327**	0.52 ± 0.09	0.405**	1.69 ± 0.09	0.150*
Splenium	0.73 ± 0.03	−0.232**	0.38 ± 0.04	0.336**	1.67 ± 0.10	0.023

Pearson’s correlations, *p < 0.05, **p < 0.01.

**Table 4.** Regression model for age and pulse pressure

Metric	Predictor	df, df_error_	SS	*F*	*p*
FA	Between subjects				
	Age	1, 170	0.161	36.246	<0.001***
	Pulse pressure	1, 170	0.007	1.552	0.215
	Within subjects				
	Subregion	4, 680	1.395	477.463	<0.001***
	Subregion x age	4, 680	0.024	8.312	<0.001***
	Subregion x pulse pressure	4, 680	0.004	1.225	0.299
RD	Between subjects				
	Age	1, 170	0.450	43.503	<0.001***
	Pulse pressure	1, 170	0.052	5.049	0.026*
	Within subjects				
	Subregion	4, 680	2.217	395.128	<0.001***
	Subregion x age	4, 680	0.066	11.761	<0.001***
	Subregion x pulse pressure	4, 680	0.005	0.893	0.433
AD	Between subjects				
	Age	1, 169	0.039	1.990	0.160
	Pulse pressure	1, 169	0.020	1.044	0.308
	Age x pulse pressure	1, 169	0.187	9.662	0.002*
	Within subjects				
	Subregion	4, 676	1.299	104.052	<0.001***
	Subregion x age	4, 676	0.026	2.076	0.082
	Subregion x pulse pressure	4, 676	0.006	0.506	0.672
	Subregion x age x pulse pressure	4, 676	0.001	0.072	0.991

df, degrees of freedom; SS, sum of squares; FA, fractional anisotropy; RD, radial diffusivity; AD, axial diffusivity.

* *p* < 0.05, **p < 0.01, ***p < 0.001.

## Results

### Effects of age on regional CC tract diffusion metrics

In the three GLMs with age as the sole between-subjects independent variable, we observed significant between-subjects main effects of age, with lower FA (*F*_(1,171)_ = 77.87, *p* < 0.0001), higher RD (*F*_(1,171)_ = 105.90, *p* < 0.0001) and higher AD (*F*_(1,171)_ = 10.24, *p* = 0.002, with increasing age. There were also significant within-subjects main effects of ROI for FA (*F*_(4,684)_ = 476.80, *p* < 0.001), RD (*F*_(4,684)_ = 395.32, *p* < 0.001), and AD (*F*_(4,684)_ = 159.03, *p* < 0.001), which indicated these diffusion metrics differed among the segments. However, this was qualified by significant age–ROI interactions for FA (*F*_(4,684)_ = 15.58, *p* < 0.0001), RD (*F*_(4,684) _= 22.96, *p* < 0.0001), and AD (*F*_(4,684)_ = 5.82, *p* < 0.0001) indicating a differential effect of age on diffusion metrics across the segments (Table 2).

**Figure 2.**
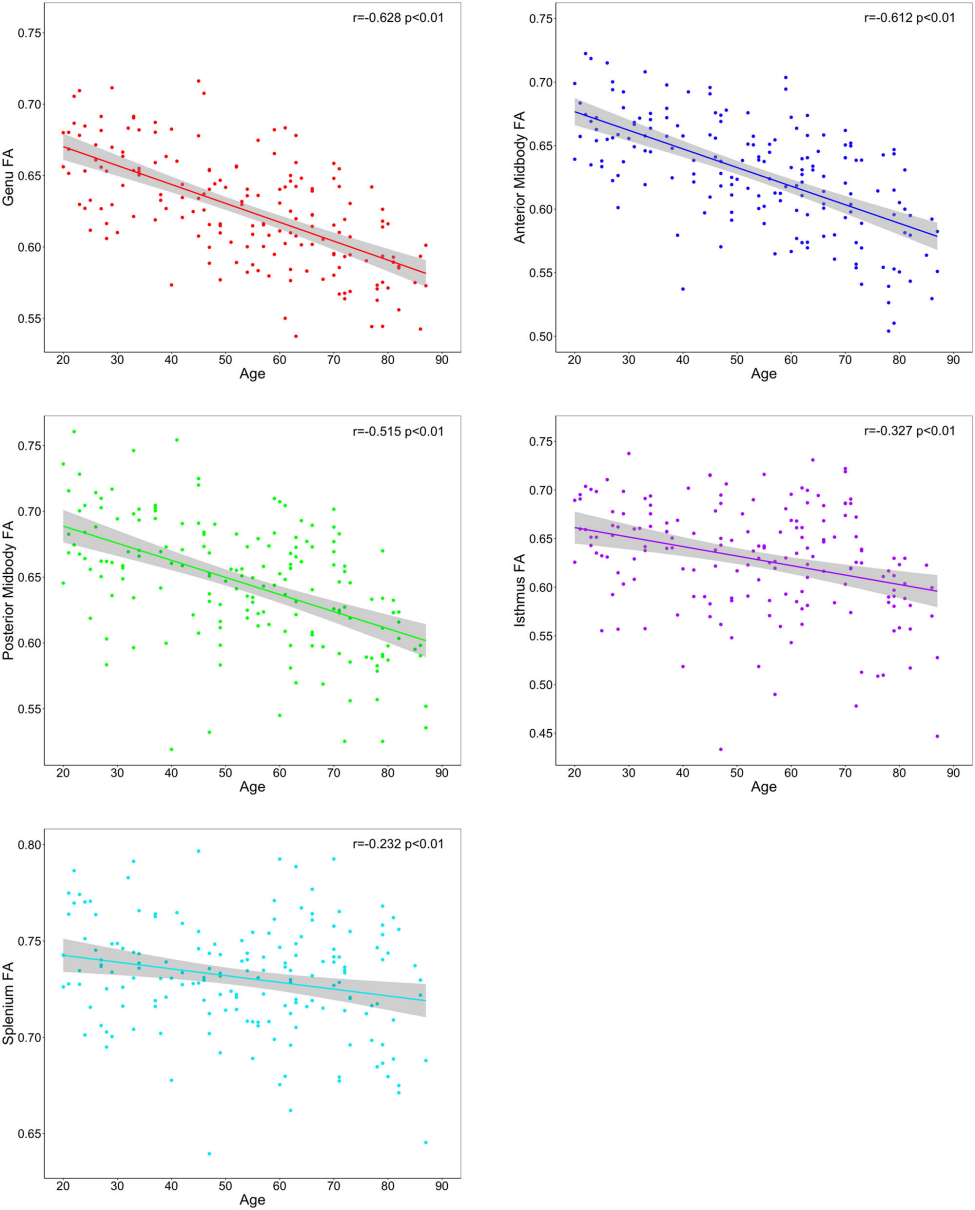
Effects of age on regional tract FA across the corpus callosum segments.

**Figure 3.**
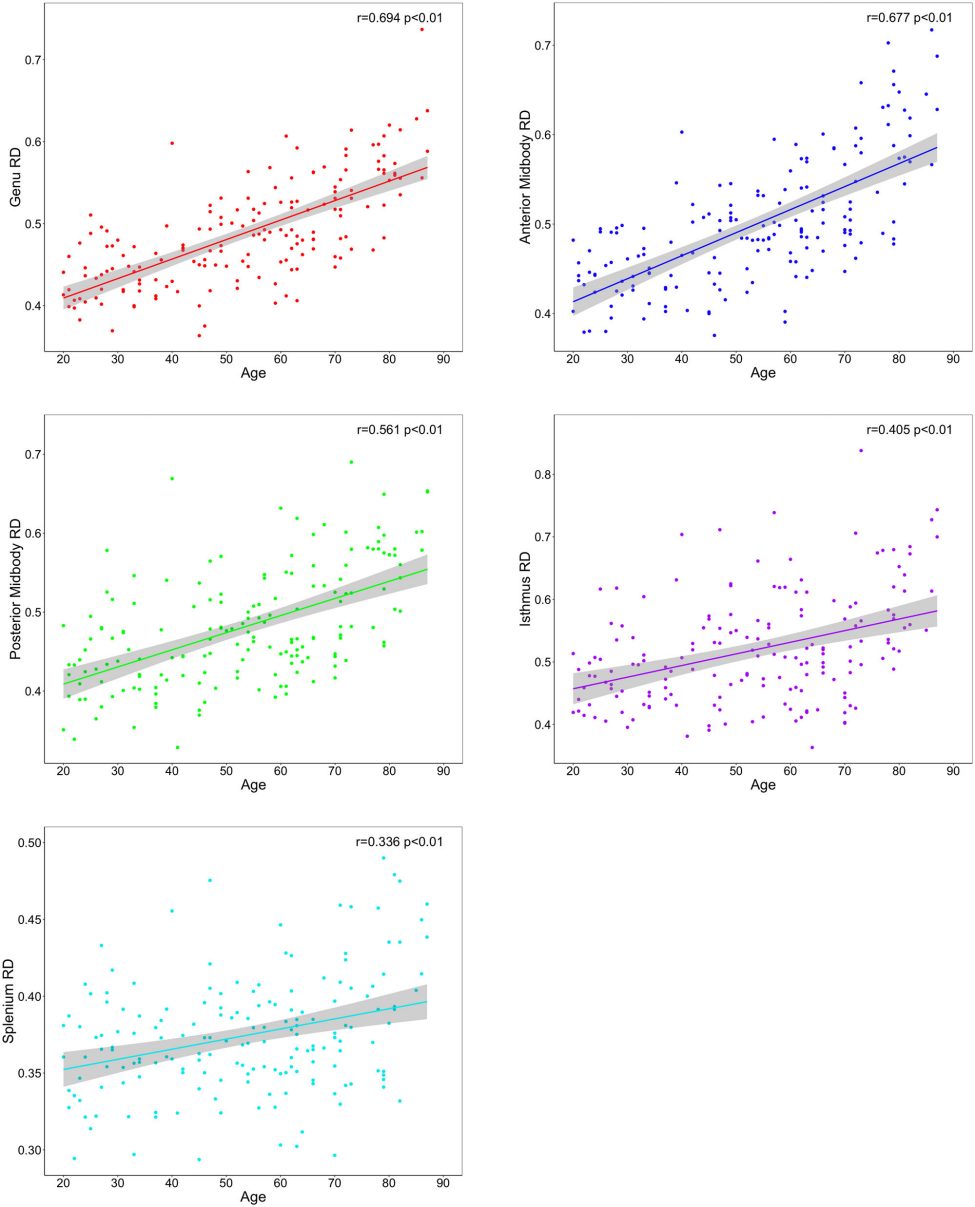
Effects of age on regional tract RD across corpus callosum segments.

**Figure 4.**
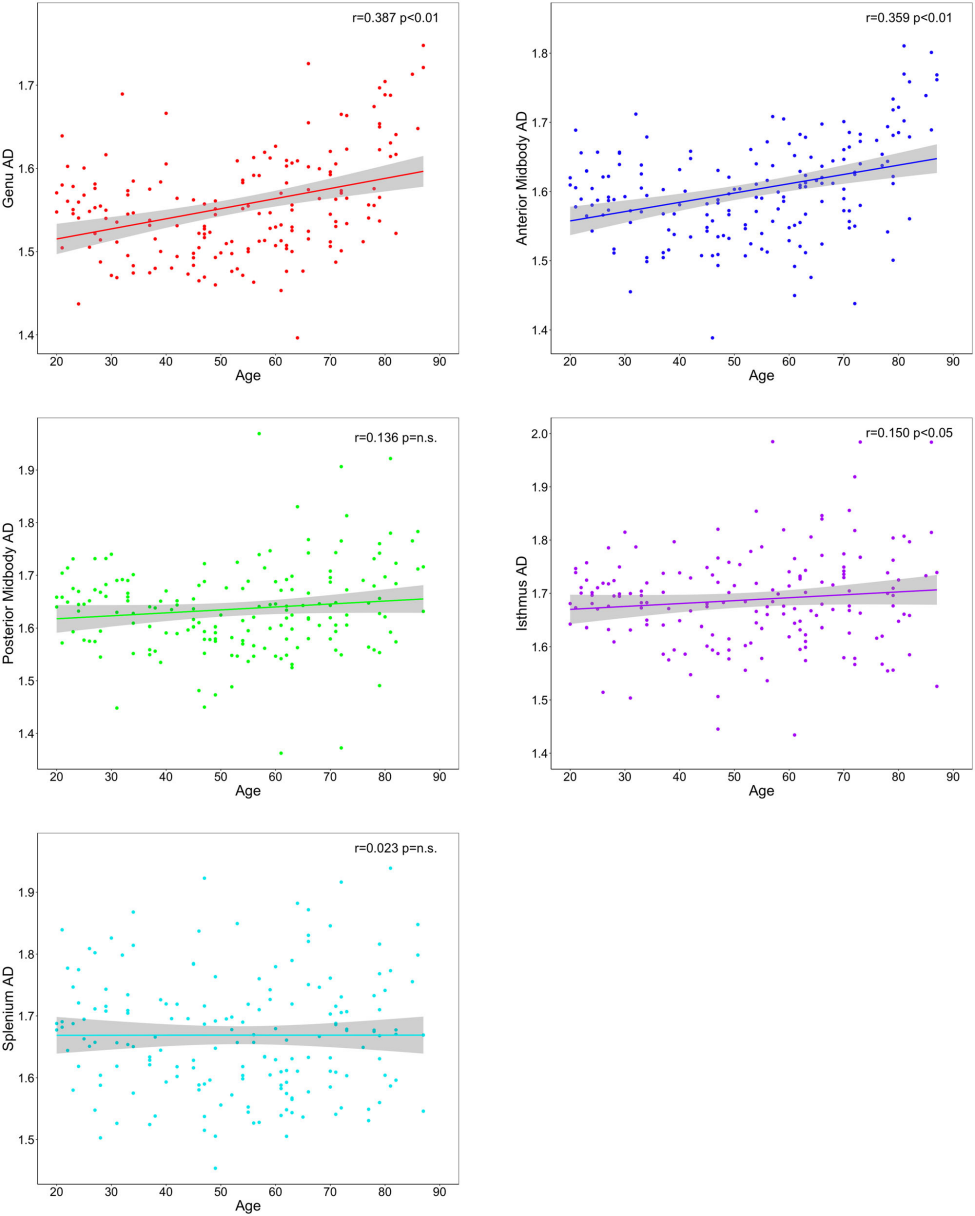
Effects of age on regional tract AD across corpus callosum segments.

To decompose the age–ROI effect, we produced zero-order correlations for the association between age and mean FA, RD, and AD values in each subregion (Table 3). The results showed a significant association between older age and lower FA values (Fig. 2) and higher RD values (Fig. 3) in every segment of the corpus callosum. Older age was also associated with higher AD values in the genu, anterior midbody, and isthmus of the corpus callosum (Fig. 4).

**Figure 5.**
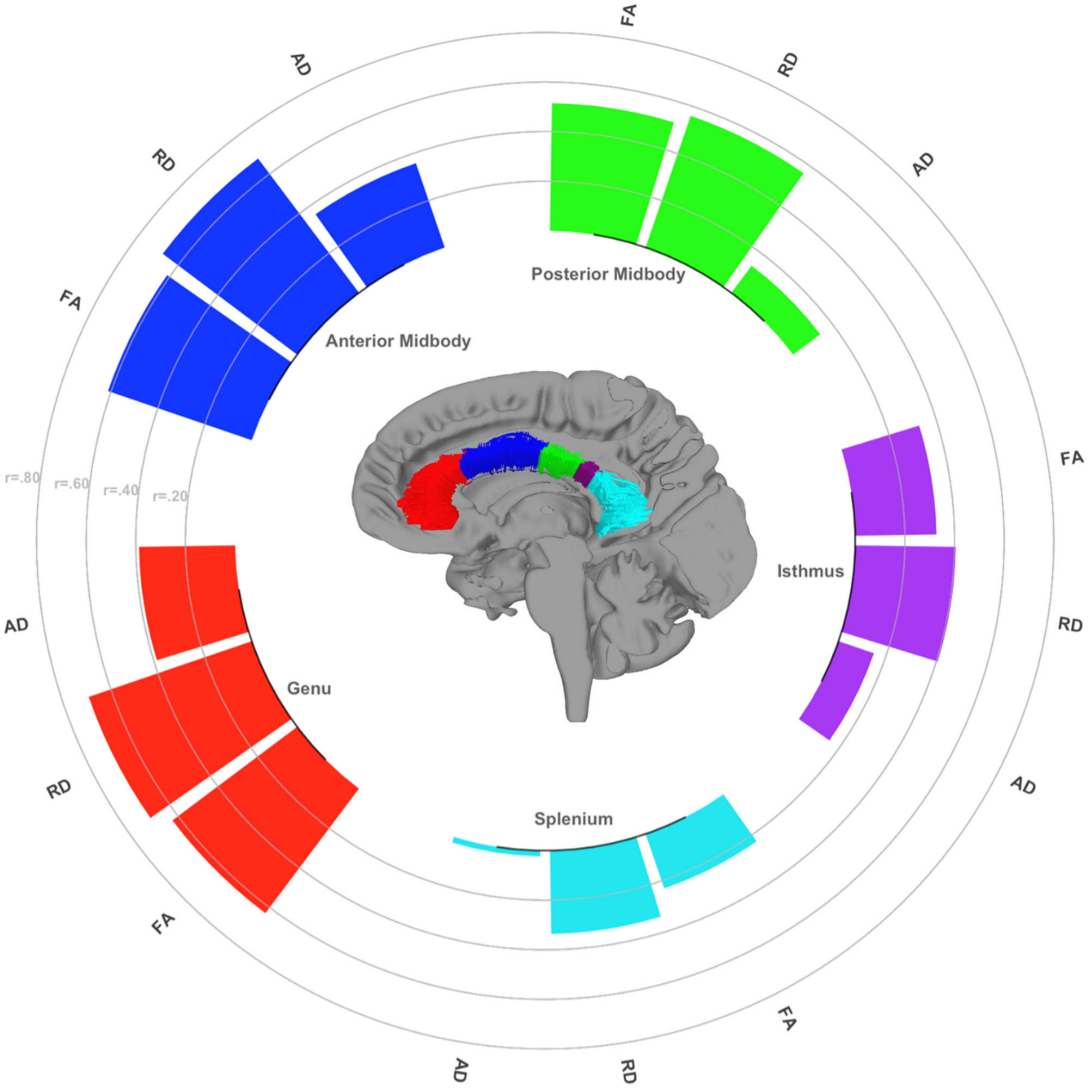
Circular barplot depicting an anteroposterior gradient of vulnerability to age across CC subregion tracts. Bar heights indicate the age–diffusion correlation for each metric, in each CC segment. Significant differences in age–diffusion associations were tested using Steiger’s *Z* tests. These associations differed significantly for FA between the genu and splenium, the genu and isthmus, the anterior midbody and splenium, the anterior midbody and isthmus, the posterior midbody and splenium (Steiger’s Z *p* < 0.001), and the posterior midbody and isthmus (*p* < 0.05). A similar stepwise pattern was found for RD: between the genu and isthmus, genu and splenium, anterior midbody and isthmus, anterior midbody and splenium, posterior midbody and splenium (*p*’s < 0.001), genu and posterior midbody, and isthmus and splenium (*p*’s < 0.05). Although weaker, this pattern exists for AD: between the genu and the splenium, the genu and the posterior midbody, the anterior midbody and splenium (*p*’s < 0.001), between the genu and isthmus, anterior midbody and isthmus, and anterior midbody and splenium (*p*’s < 0.05).

Visual inspection of Figures 2–4 suggests differences in the strength of the age metric slope across regions. To empirically compare the magnitude of age effects across CC regions, we used Steiger's *Z* test. The circular barplot in Figure 5 illustrates the differential impact of age on regions of the CC for FA, RD, and AD values. Overall, for all DTI metrics, the correlation with age was strongest in the genu and weaker in more posterior subregions in a stepwise fashion across the callosum. This anteroposterior gradient appears to be the strongest for FA and RD metrics and weakest for AD.

### Effects of pulse pressure on regional CC tract metrics

**Figure 6.**
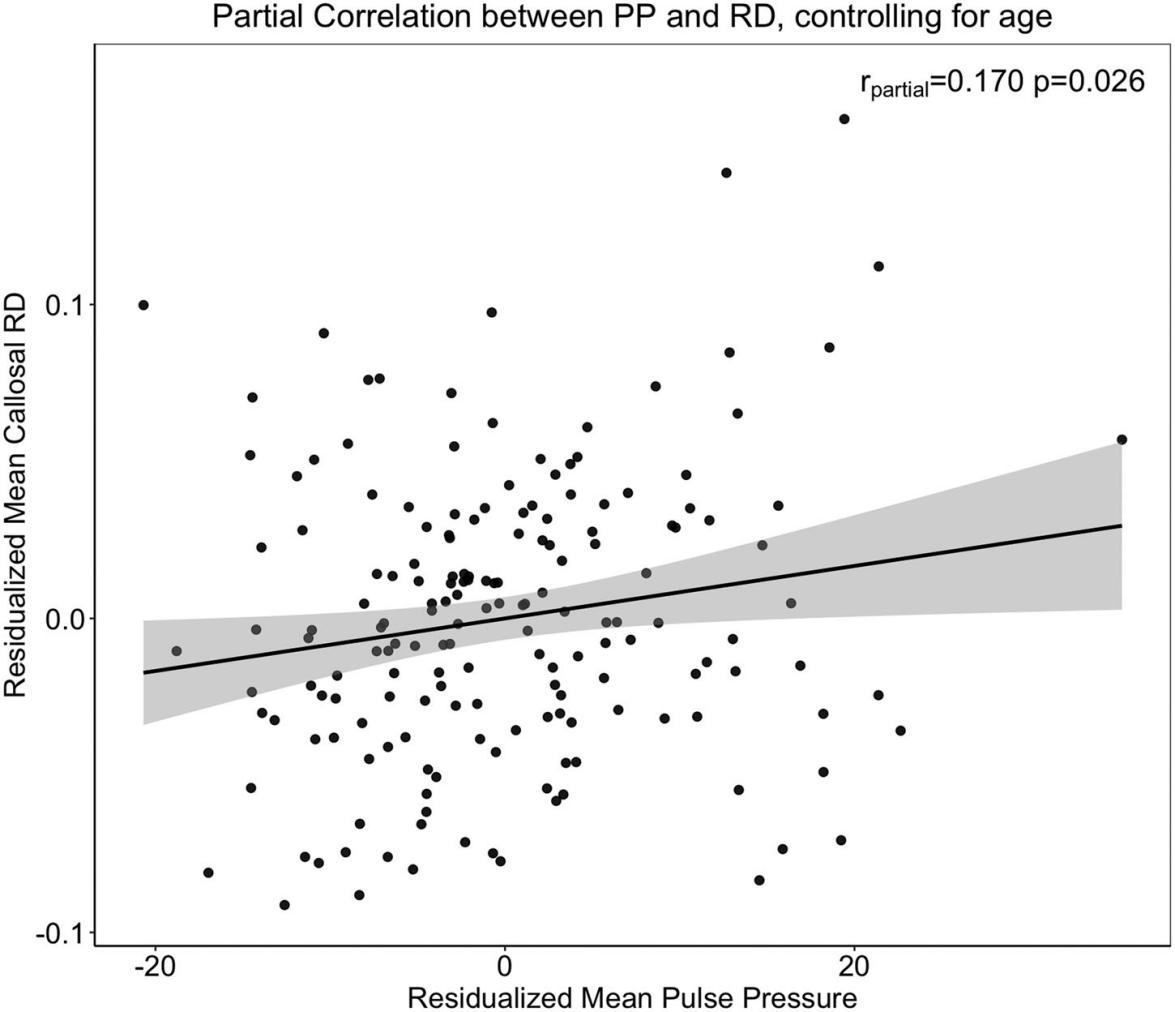
A significant association between pulse pressure and radial diffusivity, controlling for age. Radial diffusivity across the corpus callosum segments combined increases as a function of increasing pulse pressure, beyond the effects of age on RD.

To assess how pulse pressure relates to FA, AD, and RD metrics across corpus callosum subregions, GLMs were computed using pulse pressure levels and age (centered at the mean as continuous variables) and their interaction as between-subjects factors and ROI as a within-subjects dependent factor for each of the three diffusion metrics. In these models, the main effects of age on FA (*F*_(1,170)_ = 32.25, *p* < 0.001) and RD (*F*_(1,170)_ = 43.50, *p* < 0.0001), but not AD (*p* = 0.16), were found. A significant main effect of pulse pressure on RD (*F*_(1,170)_ = 5.05, *p* = 0.026), with increasing pressure associated with increasing diffusivity, was also detected. A significant age–pulse pressure interaction was observed for AD (*F*_(1,169)_ = 9.66, *p* = 0.002). Within subjects, the age–ROI interaction observed previously in the age-only models remained significant for FA (*F*_(4,680)_ = 8.31, *p* < 0.0001) and RD (*F*_(4,680)_ = 11.76, *p* < 0.0001), but not AD (*p* = 0.67; Table 4). Pulse pressure by ROI showed no reliable effects for any of the three diffusion metrics (all *p*'s > 0.3). As the effects of pulse pressure were not regionally selective, we used mean values of RD and AD across the entire corpus callosum for our secondary analyses. When age was parceled out of the association between PP and RD, the association remained significant [*r*_partial_(172) = 0.17, *p* = 0.026; Fig. 6].

**Figure 7.**
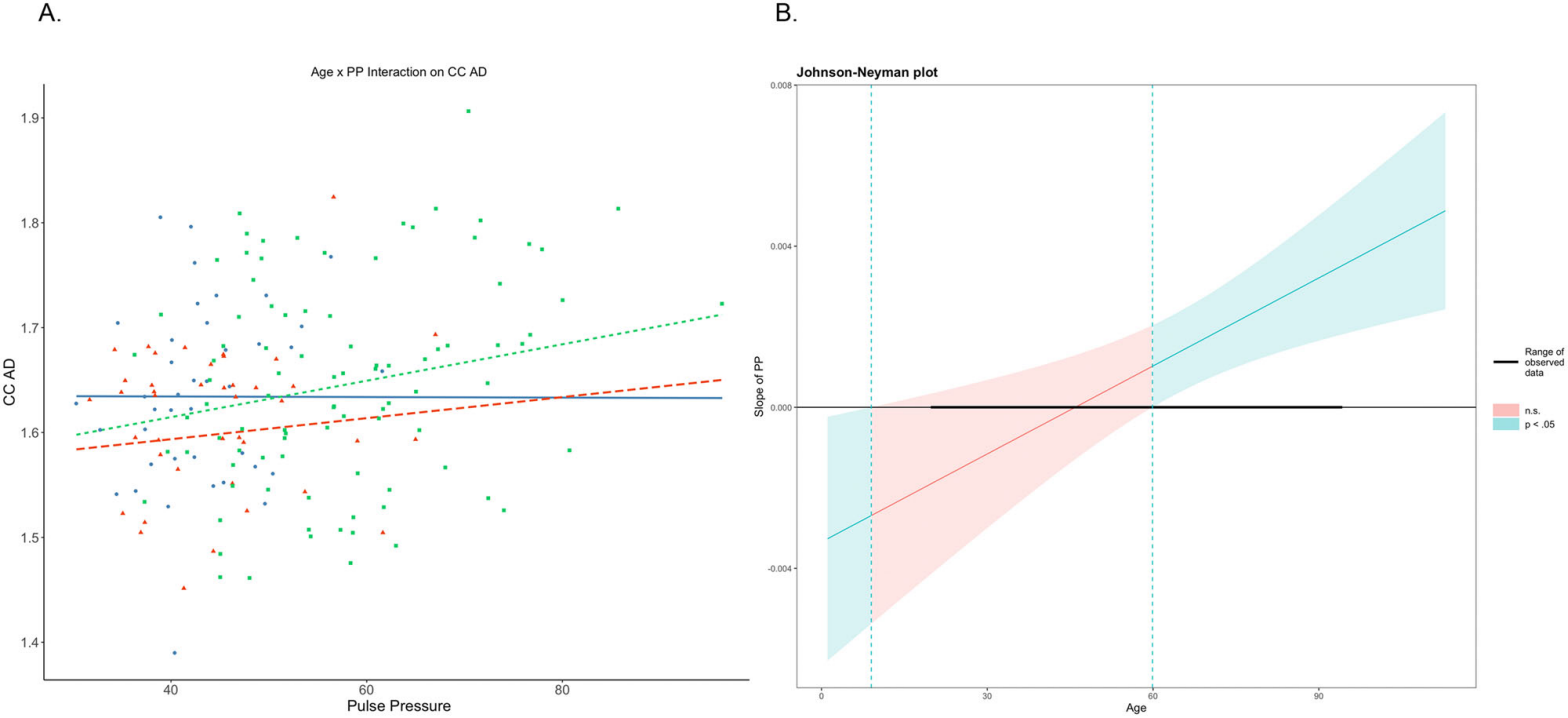
Age–pulse pressure interaction on CC axial diffusivity. ***A***, Simple slope plots illustrating the effects of pulse pressure on AD are dependent on age, with significance in middle-aged and older adults, where increasing pulse pressure is associated with increased axial diffusivity in the corpus callosum. ***B***, Johnson–Neyman interval plot suggests that the significance range of the interaction begins at approximately the age of 60 years.

To break down the interaction between age and pulse pressure on AD, we used the Johnson–Neyman method with simple slopes for visualization. Slopes were estimated at three levels: −1 standard deviation (SD) below the mean (34 years old), at the mean age (53 years old), and +1 SD above the mean (54 years old; Fig. 7). Decomposition of the interaction reveals that the association between age and pulse pressure is primarily driven by the older adults (i.e., those +1 SD above the mean), *r*(172) = 0.30, *p* = 0.01, illustrated in Figure 7*a*. Johnson–Neyman interval calculation suggests that the age–pulse pressure interaction on AD is significant for participants beginning around the age of ∼60 years old (Fig. 7*b*).

### Effects of hypertension on regional CC tract metrics

For the GLMs investigating hypertension, there remained significant effects of age on the three metrics. We found no significant main effects of hypertensive status on any of the three diffusivity metrics; however, a weak trend for RD (*p* = 0.089) was detected. The within-subjects effects of ROI by age remained significant for all three metrics (all *p*'s > 0.001). There were no significant regions by hypertension status interactions (all *p*'s > 0.26; Table 5).

**Table 5.** Regression model for age and hypertension

Metric	Predictor	df, df_error_	SS	*F*	*p*
FA	Between subjects				
	Age	1, 170	0.223	50.505	<0.001***
	Hypertension status	1, 170	0.010	2.150	0.144
	Within subjects				
	Subregion	4, 680	0.739	253.190	<0.001***
	Subregion–age	4, 680	0.026	9.074	<0.001***
	Subregion–hypertension status	4, 680	0.004	1.328	0.258
RD	Between subjects				
	Age	1, 170	0.724	69.037	<0.001***
	Hypertension status	1, 170	0.031	2.919	0.089
	Within subjects				
	Subregion	4, 680	1.162	207.338	<0.001***
	Subregion–age	4, 680	0.083	14.868	<0.001***
	Subregion–hypertension status	4, 680	0.007	1.171	0.318
AD	Between subjects				
	Age	1, 170	0.104	4.977	0.027*
	Hypertension status	1, 170	0.033	1.572	0.212
	Within subjects				
	Subregion	4, 680	1.053	84.801	<0.001***
	Subregion–age	4, 680	0.068	5.506	0.001**
	Subregion–hypertension status	4, 680	0.008	0.658	0.621

df, degrees of freedom; SS, sum of squares; FA, fractional anisotropy; RD, radial diffusivity; AD, axial diffusivity.

* *p* < 0.05, **p < 0.01, ***p < 0.001.

## Discussion

In the present study, we replicate findings that the white matter fibers of the corpus callosum are highly vulnerable to the aging process. Moreover, our results demonstrate that the callosal subregions are differentially sensitive to age-related deterioration, as the strength of the associations between age and FA uniformly decrease from genu to splenium, echoing the anteroposterior gradient observed in white matter across the brain (Davis et al., 2009; Kennedy and Raz, 2009b; Bennett et al., 2010). According to the “last in, first out” hypothesis (Raz, 2000), this anteroposterior gradient is a reversal of the pattern of white matter development, in which prefrontal regions are among the last to complete myelination. Associations between age and RD also followed the anteroposterior gradient in our findings, and anterior “late-myelinating” subregions had the greatest percentage of variance explained by age-related changes in RD. Overall, age was more strongly associated with RD than AD, suggesting that de/dysmyelination may be more integral to normal aging processes than total axonal degeneration, which may require frank injury or pathology to the brain. Other researchers have noted the opposite (Vernooij et al., 2008) and attributed white matter changes primarily to axonal degeneration and loss of macrostructural white matter organization. It may be that differing patterns of RD and AD alterations denote dissociable biological aging mechanisms (Bartzokis et al., 2012) and that these different processes are temporally and regionally specific (Burzynska et al., 2010). The current study supports existing evidence that anterior regions of the corpus callosum exhibit steeper volumetric declines compared with posterior regions during late life (Hasan et al., 2009; Hsu et al., 2010).

The null finding for the effects of hypertension as a diagnostic category on callosal white matter contradicts results from several previous studies that demonstrated an influence of hypertension diagnosis on both diffusivity and lesion load in the callosum (Kennedy and Raz, 2009b; Burgman et al, 2010; Gons et al., 2012). It could be that as a dichotomous diagnostic entity, hypertension is a more general factor compared with pulse pressure, which is the difference between systolic and diastolic blood pressure and is representative of arterial stiffness in the walls of larger arteries (Franklin et al., 1999), and perhaps reflects a more specific, potentially mechanistic factor (arterial stiffness). In our study, we found that pulse pressure was associated with a negative, regionally invariant pattern of callosal microstructure. Specifically, pulse pressure appears to disrupt the anteroposterior aging gradient, as differential segment–age associations were lost when accounting for pulse pressure, suggesting that relatively age-invariant posterior regions become sensitive when cardiovascular risk is introduced. This may be attributable to de/dysmyelination in posterior subregions, as pulse pressure is selectively associated with CC isthmus and splenium for the radial diffusivity metric. Additionally, we found that the interaction between age and pulse pressure on axial diffusivity becomes significant at approximately the age of 60 years (Fig. 7*b*). These findings suggest that pulse pressure begins to impact white matter health in the callosum by late midlife/early old age. We note that it is still a matter of debate and ongoing investigation of what biological proxies the different diffusion metrics might reflect (Petiet et al., 2019; Dhiman et al., 2022).

Our findings align with those of Vemuri and colleagues who found that existing cardiovascular and metabolic conditions in an older adult sample were associated with lower FA in the corpus callosum and fornix (Vemuri et al., 2018). The results from the current study support these collective findings using a separate measure of cardiovascular health and finer-grained parcellations of the corpus callosum. Furthermore, we extend these findings throughout an adult lifespan sample. Although accumulating evidence suggests that the regions of the corpus callosum are differentially impacted by cardiovascular risk factors, the neural mechanisms underlying cardiovascular health's influence on white matter are not well understood. Extant literature points to reduced cerebral blood perfusion and ischemic events as possible mechanistic factors (Jefferson et al., 2011). Whatever mechanisms are responsible, these cardiovascular-related white matter alterations may represent a form of pathological aging, as poor cardiovascular health is considered a major risk factor for vascular dementia (O’Brien and Thomas, 2015), and, to a lesser degree, Alzheimer’s disease (Rodrigue et al., 2013).

A longer duration of hypertension diagnosis has been associated with greater age-related deterioration of posterior white matter areas, whereas elevations in pulse pressure, in a solely normotensive population, were linked to greater deterioration in anterior regions only (Kennedy and Raz (2009b) and replicated in other studies (Maillard et al., 2012; Salat et al., 2012; VandeBunte et al., 2023). Small increments in blood pressure levels may exacerbate age effects in the already compromised anterior subregions and, at higher levels, induce them in the relatively preserved posterior subregions, thereby disrupting the anteroposterior gradient in typical aging.

The corpus callosum plays a critical role in cognition, and metrics of callosal microstructure integrity are linked to better cognitive performance in working memory, processing speed, and executive functioning (Madden et al., 2004; Kennedy and Raz, 2009a; Alioto et al., 2019). However, we have demonstrated regional variability in callosal aging, and combined with the modifying influence of cardio- and cerebrovascular health, these findings may have implications for specific cognitive functions that are thought to be dissociable for callosal individual subregions. Executive functions associated with the prefrontal connections through the genu are among the cognitive abilities most sensitive to age; declines in genu FA, but not splenium FA, are related to poorer cognition (Kennedy and Raz, 2009a; Hoagey et al., 2021; VandeBunte et al., 2023). In contrast, motor coordination is solely associated with FA in the body of the callosum (Johansen-Berg et al., 2007), which connects the premotor in the anterior body and the primary motor through the posterior midbody. These dissociations are due to the fact that mainly unimodal cortical projections pass through each subregion (Hofer and Frahm, 2006). In the presence of cardiovascular risk factors, cognitive functions mediated by anterior regions may face even steeper declines and may have implications for MCI and AD. Indeed, cardiovascular risk factors have been associated with degradations in white matter microstructure, and carrying an APOE e4+ genotype exacerbates both cognitive decline and white matter deterioration (Wang et al., 2015). Accumulating evidence suggests that white matter integrity specifically within the genu of the CC predicts cognitive decline and subsequent mild cognitive impairment diagnosis, even after controlling for AD biomarkers (Raghavan et al., 2020). Future research should explore the impact of cardiovascular health interventions on susceptibility to callosal microstructure degradation across the lifespan. For example, one way that exercise positively affects brain health and cognition is by increasing cerebral blood flow (Tarumi et al., 2022; Tomoto et al., 2023).

There are several limitations to the study that must be considered. Given that this is a cross-sectional report, further longitudinal investigations are needed to verify whether the patterns of aging present in our findings are representative of true within-person changes in aging. Such data are currently being collected in follow-up waves to this study. The current study, while focused on the effects of pulse pressure and hypertension, was not sufficiently powered to control or test for the effects of antihypertensive medication use. Future studies with sufficient sample sizes should assess the impact that antihypertensive medication may have on white matter integrity in the corpus callosum throughout the lifespan. On a related note, this study primarily focused on the impact of pulse pressure and hypertension, given that these are some of the most direct, feasibly collectible markers of heart function (Thomas et al., 2013; Boutouyrie et al., 2021). It should be emphasized, however, that these are only two among several cardiovascular factors that may impact white matter integrity. Both cholesterol (Kang et al., 2023) and metabolic diseases, such as diabetes (Tamura and Araki, 2015), are associated with white matter degradation and the accumulation of white matter hyperintensities. The current study did not include a collection of blood samples to measure cholesterol levels. Furthermore, the sample deliberately excluded individuals with diabetes and other metabolic disease in order to study the effects of healthy aging on the brain, minimizing disease-related confounds. Future research should investigate the multifactorial impact of cardiac and metabolic diseases to assess how multiple comorbidities impact callosal white matter. Additionally, diffusion tensor imaging carries its own limitations (Jones and Cercignani, 2010; Figley et al., 2022), although careful quality checks, visual inspection of all images, and proper tractography methods utilized in this study were applied to help mitigate those limitations. Many of the limitations in diffusion tensor models are most notable in complex white matter areas of intermixing fiber types and less applicable to the fibers comprising the corpus callosum.

In conclusion, aging differentially and negatively impacts the microstructural health of the subregions of the corpus callosum, and elevations in pulse pressure may alter these patterns of aging, even in individuals without diagnosed hypertension. Maintaining optimal blood pressure levels to avoid or delay age-related arterial stiffening could be a key factor to successful aging, but more research is needed in this area to determine how cerebro- and cardiovascular health interact with age to influence brain structure and cognition.

## Synthesis

Reviewing Editor: Alexander Soutschek, Ludwig-Maximilians-Universitat Munchen Decisions are customarily a result of the Reviewing Editor and the peer reviewers coming together and discussing their recommendations until a consensus is reached. When revisions are invited, a fact-based synthesis statement explaining their decision and outlining what is needed to prepare a revision will be listed below. The following reviewer(s) agreed to reveal their identity: Kaitlin Casaletto, William Hopkins.

Reviewer #1

Manuscript eneuro-562251-31102023 broadly examines differential aging effects on regional corpus callosum microstructure in a lifespan cohort. They further explore whether these effects are moderated by blood pressure. This work is particularly relevant given prior literature supporting blood pressure as a highly important modifiable risk factor for cognitive aging. This manuscript was clearly written and had clear aims and objectives, and the analyses conducted appear appropriate. However, we suggest including further clarification in several sections of the paper, and further explanation in the introduction, results, and discussion. See below for more specific feedback.

General Comments

- This paper is unique in that it is exploring an important question for cognitive aging and dementia in a lifespan cohort. We think this is exciting work and the authors should highlight this more throughout their paper.

- Throughout the paper, the authors indicate "vascular" without specification of whether they mean cardiovascular versus cerebrovascular. Given the importance of this study's aim to clarify whether a cardiovascular indicator (blood pressure) contributes to cerebrovascular health (corpus collosum white matter integrity), we suggest clearly stating cardiovascular or cerebrovascular, as appropriate. We have pointed out several instances here, but please consider changing throughout the manuscript.

o Abstract: Lines 16 and 19 (vascular = cardiovascular)

o Introduction: Lines 38, 80, 85, 88, 91 (vascular = cardiovascular)

Abstract

- Please clearly state and acknowledge all the measures used in the study (pulse systolic versus diastolic hypertension). You may also consider adding in the calculation for pulse pressure in the abstract.

- We suggest the authors highlight the use of a lifespan cohort in their study and clearly indicate that they are running cross-sectional analyses.

Introduction

- Please provide clearer justification for how the authors selected and operationalized cardiovascular health (e.g., why pulse pressure versus other indicators of cardiovascular health?).

o Relatedly, please include a description of pulse pressure and the relevant literature supporting pulse pressure as an appropriate metric for systemic cardiovascular health.

- As the authors outline in their introduction, there are several studies that have already examined associations between age and regionality of the corpus collosum, along with regionality of cardiovascular risk and cerebrovascular health. We suggest the authors clearly define the gap in the literature that they are intending to fill. For example, what is different in this study than other previous works (e.g., lifespan cohort)? What about their study is similar to other prior works? Alternatively, if this manuscript is intended to be a replication paper, please explicitly state that.

- The authors discuss how cardiovascular (assuming vascular = cardiovascular, line 80) health impacts white matter integrity. There are several works connecting cardiovascular health to white matter health, and we suggest the authors broaden the literature on this topic before narrowing in on blood pressure. From there, the authors may choose to then highlight what led them to use blood pressure as a systemic cardiovascular health metric.

- In lines 85-90, the authors discuss mixed literature on regionality of the corpus collosum, highlighting several papers that were published 10+ years ago. Regionality of the corpus collosum has been lighted in recent works that the authors may consider including:

o https://pubmed.ncbi.nlm.nih.gov/36710073/

o https://pubmed.ncbi.nlm.nih.gov/33022474/

o https://pubmed.ncbi.nlm.nih.gov/30264411/

- While the authors nicely outlined the goal of the study, we suggest they take it a step further and add some additional information on their hypotheses. For example, do the authors postulate that cardiovascular health is a mechanism through which age and regional corpus collosum are related? If so, please clearly state this as an aim of the study.

Methods

- Please provide justification on why pulse pressure was chosen as a metric of cardiovascular health somewhere in the methods section (if the authors have not already done so in the introduction).

Results

- Please consider including a statistical analysis section to increase transparency. For example, the following things were not clear:

o Were the age effects conducted in an independent model or was the main effect interpreted in the context of an interaction model?

o What were the covariates for each model?

- The overarching goals of the moderation analyses were not entirely clear. Based on the rationale in the Introduction, it appeared the authors were considering declines in cardiovascular health as a mechanism linking age to white matter changes. If so, they might consider conducting a mediation model versus a moderation (eg, first examine associations between age and cardiovascular health indicators factors, and then run a mediation model). This would allow the authors to examine which ages across the lifespan these cardiovascular indicators start to explain the relationship between age and CC integrity.

- A table that summarized the regression models with all parameters would significantly increase interpretation of the findings.

- In the hypertension results section (lines 246-248), the authors only provide one overarching statement on their lack of findings with this cardiovascular health indicator. Additional description of the models conducted (even if not reaching statistical significance) is important. Further, we feel there may be several additional analyses to further probe this relationship that could have important implications for the discussion. For example, did the authors test for effects of antihypertensive medication? If not available, this is a limitation and should be clearly outlined and then discussed in the discussion section.

Discussion

- In line 268, the authors state "non-linearity of age effects." The authors did not test for nonlinear effects of age (e.g., age^2).

- The hypertension analysis should be probed further before concluding null findings (see above).

- The authors interpret each diffusion metric as reflecting a specific aspect of white matter biology (e.g., myelination vs axon degeneration). Please acknowledge that these metrics are still being validated and there may be limitations in what each reflects.

#########################

Reviewer #2

This is a solid paper describing association between age and CC microstructure integrity and how they are mediated by cardiovascular measures which presumably impact white matter morphology. It provides novel results and therefore advances the field.

I have some concerns in the decision to remove sex a predictor variable in all the models. No sex differences were reported and therefore this variable was excluded in subsequent analyses. I understand why the authors then removed this variable in subsequent analyses but I am curious to know if sex remains in the statistical models, are the effects still evident?

Also, in some of the GLM models that included, ROI as a variable, 7 Is the numerator degree of freedom but that seems wrong to me unless I am missing some thing in the treatment and levels of the predictor variables. Can this be clarified? Also, are the GLM model, is ROI treated as a within subject variable? From some of the analyses, this is clearly stated but not in the initial sets of analyses.

The FA, RD and AD measures were not adjusted for whole brain or lobular values. That said, can the authors really make the case that these effects may or may not be specific to the CC or reflect a more global overall effects of the brain? Indeed, in light of the fact that the termination of fibers traversing the CC progress along a anterior to posterior gradient, it seems that adjustment in CC values would ideally ben] done for frontal, parietal, temporal and occipital regions.

## Author Response

Dear Dr. Soutschek, We appreciate the opportunity to revise our manuscript to clarify and improve the writing. We respond point-by-point to the two reviewer's thoughtful comments in turn below. The reviewer's comments are in italics and our responses are in blue font, nonitalicized. In the manuscript text, all changes appear in yellow highlight.

Reviewer #1: "Manuscript eneuro-562251-31102023 broadly examines differential aging effects on regional corpus callosum microstructure in a lifespan cohort. They further explore whether these effects are moderated by blood pressure. This work is particularly relevant given prior literature supporting blood pressure as a highly important modifiable risk factor for cognitive aging. This manuscript was clearly written and had clear aims and objectives, and the analyses conducted appear appropriate. However, we suggest including further clarification in several sections of the paper, and further explanation in the introduction, results, and discussion. See below for more specific feedback." General Comments - This paper is unique in that it is exploring an important question for cognitive aging and dementia in a lifespan cohort. We think this is exciting work and the authors should highlight this more throughout their paper.

We thank the reviewer for their kind suggestions. We have emphasized the lifespan cohort further throughout our paper.

- Throughout the paper, the authors indicate "vascular" without specification of whether they mean cardiovascular versus cerebrovascular. Given the importance of this study's aim to clarify whether a cardiovascular indicator (blood pressure) contributes to cerebrovascular health (corpus collosum white matter integrity), we suggest clearly stating cardiovascular or cerebrovascular, as appropriate. We have pointed out several instances here, but please consider changing throughout the manuscript. o Abstract: Lines 16 and 19 (vascular -> cardiovascular) o Introduction: Lines 38, 80, 85, 88, 91 (vascular -> cardiovascular) These specific changes have been made, as well as other instances throughout the manuscript.

Abstract - Please clearly state and acknowledge all the measures used in the study (pulse systolic versus diastolic hypertension). You may also consider adding in the calculation for pulse pressure in the abstract.

This has been addressed.

- We suggest the authors highlight the use of a lifespan cohort in their study and clearly indicate that they are running cross-sectional analyses.

This has been addressed.

Introduction - Please provide clearer justification for how the authors selected and operationalized cardiovascular health (e.g., why pulse pressure versus other indicators of cardiovascular health?).Relatedly, please include a description of pulse pressure and the relevant literature supporting pulse pressure as an appropriate metric for systemic cardiovascular health.

We thank the reviewer for their suggestion to strengthen the article. Discussion regarding the utility of pulse pressure as a metric of cardiovascular health has been added, beginning on line 100.

- As the authors outline in their introduction, there are several studies that have already examined associations between age and regionality of the corpus collosum, along with regionality of cardiovascular risk and cerebrovascular health. We suggest the authors clearly define the gap in the literature that they are intending to fill. For example, what is different in this study than other previous works (e.g., lifespan cohort)? What about their study is similar to other prior works? Alternatively, if this manuscript is intended to be a replication paper, please explicitly state that.

We have added to our study motivation paragraph (beginning on line 104) the gaps in the literature that this study sought to fill (the lack of studies sampling middle aged and younger adults and the lack of studies with inclusion criteria that excludes frank diseases).

- The authors discuss how cardiovascular (assuming vascular = cardiovascular, line 80) health impacts white matter integrity. There are several works connecting cardiovascular health to white matter health, and we suggest the authors broaden the literature on this topic before narrowing in on blood pressure. From there, the authors may choose to then highlight what led them to use blood pressure as a systemic cardiovascular health metric.

We have now edited this section, including more broader general associations before narrowing to our focus (line 80 on).

- In lines 85-90, the authors discuss mixed literature on regionality of the corpus collosum, highlighting several papers that were published 10+ years ago. Regionality of the corpus collosum has been lighted in recent works that the authors may consider including: o https://pubmed.ncbi.nlm.nih.gov/36710073/ o https://pubmed.ncbi.nlm.nih.gov/33022474/ o https://pubmed.ncbi.nlm.nih.gov/30264411/ We thank the reviewer for their suggestion in including more recent works. The following recent studies (and others) have been added to the discussion section of the manuscript (beginning on lines 333).

- While the authors nicely outlined the goal of the study, we suggest they take it a step further and add some additional information on their hypotheses. For example, do the authors postulate that cardiovascular health is a mechanism through which age and regional corpus collosum are related? If so, please clearly state this as an aim of the study.

We have now made our study goals and hypotheses more specific, beginning on line 128. We tend to not use the term mechanistic in our work because we feel it has too causal of a connotation than we are comfortable with, but we think we understand what the reviewers are thinking and so clarified that we are testing for synergistic effects of age and cardiovascular factors (i.e., statistical moderation) and the differential regional effects. (Line131) Methods - Please provide justification on why pulse pressure was chosen as a metric of cardiovascular health somewhere in the methods section (if the authors have not already done so in the introduction).

Justification for the use of pulse pressure has been included in the introduction, beginning on line 100 Results - Please consider including a statistical analysis section to increase transparency. For example, the following things were not clear: o Were the age effects conducted in an independent model or was the main effect interpreted in the context of an interaction model? o What were the covariates for each model? We agree that the data analysis plan was not obvious as written. We now include a statistical analysis section outlining the models to be run and the variables included in the models (beginning on line 201).

- The overarching goals of the moderation analyses were not entirely clear. Based on the rationale in the Introduction, it appeared the authors were considering declines in cardiovascular health as a mechanism linking age to white matter changes. If so, they might consider conducting a mediation model versus a moderation (eg, first examine associations between age and cardiovascular health indicators factors, and then run a mediation model). This would allow the authors to examine which ages across the lifespan these cardiovascular indicators start to explain the relationship between age and CC integrity.

We believe that we address this point in the reviewer's point above regarding clarifying the hypotheses.

- A table that summarized the regression models with all parameters would significantly increase interpretation of the findings.

We have added tables to provide the source tables of our model output (Tables 2, 4, and 5).

- In the hypertension results section (lines 246-248), the authors only provide one overarching statement on their lack of findings with this cardiovascular health indicator. Additional description of the models conducted (even if not reaching statistical significance) is important. Further, we feel there may be several additional analyses to further probe this relationship that could have important implications for the discussion. For example, did the authors test for effects of antihypertensive medication? If not available, this is a limitation and should be clearly outlined and then discussed in the discussion section.

We have provided more statistics in the hypertension results section for the nonsignificant effects, and all the model terms and statistical parameters can be found in the three new Tables (one for each diffusion metric). Given our sample size, we did not feel like we had the statistical power to assess the effects of antihypertension medications, especially if we grouped medications by type. This has been added as a limitation beginning on line 380.

Discussion - In line 268, the authors state "non-linearity of age effects." The authors did not test for nonlinear effects of age (e.g., age^2).

This was a failure to remove old text. We did originally test each segment for each diffusion metric for nonlinearity, but as only one or two regions proved to be nonlinear with age in this sample, we removed that section from the results. We have completely removed these statements now.

- The hypertension analysis should be probed further before concluding null findings (see above).

More information has been provided on these models as mentioned above.

- The authors interpret each diffusion metric as reflecting a specific aspect of white matter biology (e.g., myelination vs axon degeneration). Please acknowledge that these metrics are still being validated and there may be limitations in what each reflects.

The reviewer makes a good point. We have strengthened the acknowledgement of this point in the discussion on line 331 Reviewer #2: "This is a solid paper describing association between age and CC microstructure integrity and how they are mediated by cardiovascular measures which presumably impact white matter morphology. It provides novel results and therefore advances the field." I have some concerns in the decision to remove sex a predictor variable in all the models. No sex differences were reported and therefore this variable was excluded in subsequent analyses. I understand why the authors then removed this variable in subsequent analyses but I am curious to know if sex remains in the statistical models, are the effects still evident? To double check this for the reviewer, we reran all the models with sex added back in and the patterns of results were completely unchanged. We also added this to the new statistical analysis section on line 213 Also, in some of the GLM models that included, ROI as a variable, 7 Is the numerator degree of freedom but that seems wrong to me unless I am missing some thing in the treatment and levels of the predictor variables. Can this be clarified? Also, are the GLM model, is ROI treated as a within subject variable? From some of the analyses, this is clearly stated but not in the initial sets of analyses.

We apologize for the confusion. A new statistical analysis section (beginning on line 201) and statistical model output tables have been added (Tables 2, 4 and 5) to clarify predictors. Corpus Callosum ROI was treated as a within-subject variable. Regarding df, we thank the reviewer for their careful reading of our manuscript and catching this discrepancy. We have corrected all the reporting of df now.

The FA, RD and AD measures were not adjusted for whole brain or lobular values. That said, can the authors really make the case that these effects may or may not be specific to the CC or reflect a more global overall effects of the brain? Indeed, in light of the fact that the termination of fibers traversing the CC progress along a anterior to posterior gradient, it seems that adjustment in CC values would ideally ben] done for frontal, parietal, temporal and occipital regions.

We think we are interpreting the reviewer's comment correctly: we do not think that the anterior-to-posterior gradient is selective to the corpus callosum. In fact, we have other published papers demonstrating this (and other) spatial gradients across the brain's white matter. Although not what the reviewer specifically meant, this comment did spur us to consider whether the three diffusion metrics from the five regions might be significant beyond the general aging of the whole brain. In some of our other DTI papers (where white matter indices are the independent variables predicting cognition or other dependent variables) we use whole brain FA as a covariate of no interest to demonstrate that the tract of interest in those studies were selectively associated with the dependent variable (i.e., the tract is a significant predictor of cognition rather than general white matter being a sufficient predictor). We tried to do this out of curiosity during this revision in two ways. Both appear to be overly punitive when white matter diffusion is the dependent variable in a study. We first added whole brain FA (or RD or AD) as a covariate of no interest along with the other predictors. While most, but not all of our results held, whole brain FA/RD/AD covariate was highly significant in each model (not surprisingly given the collinearity). We then regressed FA/RD/AD out of each of the 5 CC segments and then reran the models with the residualized variables instead of the raw variables, and again, while most of the findings held, a couple did not, and we decided that this was just an overly statistically punitive exercise. We thank the reviewer for sparking the idea to conduct these exercises.

We thank the editor and the two reviewers for their careful reading of our manuscript and for the helpful comments. The paper reads much more clearly now for it. We hope that this version is suitable for publication in eNeuro.

Sincerely, The authors
